# RNA‐protein interactions in an unstructured context

**DOI:** 10.1002/1873-3468.13116

**Published:** 2018-06-21

**Authors:** Bojan Zagrovic, Lukas Bartonek, Anton A. Polyansky

**Affiliations:** ^1^ Department of Structural and Computational Biology Max F. Perutz Laboratories University of Vienna Austria; ^2^ MM Shemyakin and Yu A Ovchinnikov Institute of Bioorganic Chemistry Russian Academy of Sciences Moscow Russia

**Keywords:** intrinsically disordered proteins, long noncoding RNAs, nucleobase/amino acid interaction affinity scales, RNA–protein granules, RNA–protein interactions

## Abstract

Despite their importance, our understanding of noncovalent RNA–protein interactions is incomplete. This especially concerns the binding between RNA and unstructured protein regions, a widespread class of such interactions. Here, we review the recent experimental and computational work on RNA–protein interactions in an unstructured context with a particular focus on how such interactions may be shaped by the intrinsic interaction affinities between individual nucleobases and protein side chains. Specifically, we articulate the claim that the universal genetic code reflects the binding specificity between nucleobases and protein side chains and that, in turn, the code may be seen as the Rosetta stone for understanding RNA–protein interactions in general.

## Abbreviations

**ADE,** adenine

**CYT,** cytosine

**FMRP,**Fragile X Mental Retardation Protein

**FUS,** Fused‐In‐Sarcoma

**GO,** Gene Ontology

**GUA,** guanine

**IDPs,** unstructured/disordered proteins

**lncRNAs,**long noncoding RNAs

**MD,** molecular dynamics

**PUR,** purine; PYR, pyrimidine

**RBDs,** RNA‐binding domains

**RBPs,** RNA‐binding proteins

**RBR,** RNA‐binding regions

**RF,** random forest

**RRM,** RNA recognition motif

**SVM,** support vector machine

**URA,** uracil

**UTRs,** untranslated regions

**Figure 1:**
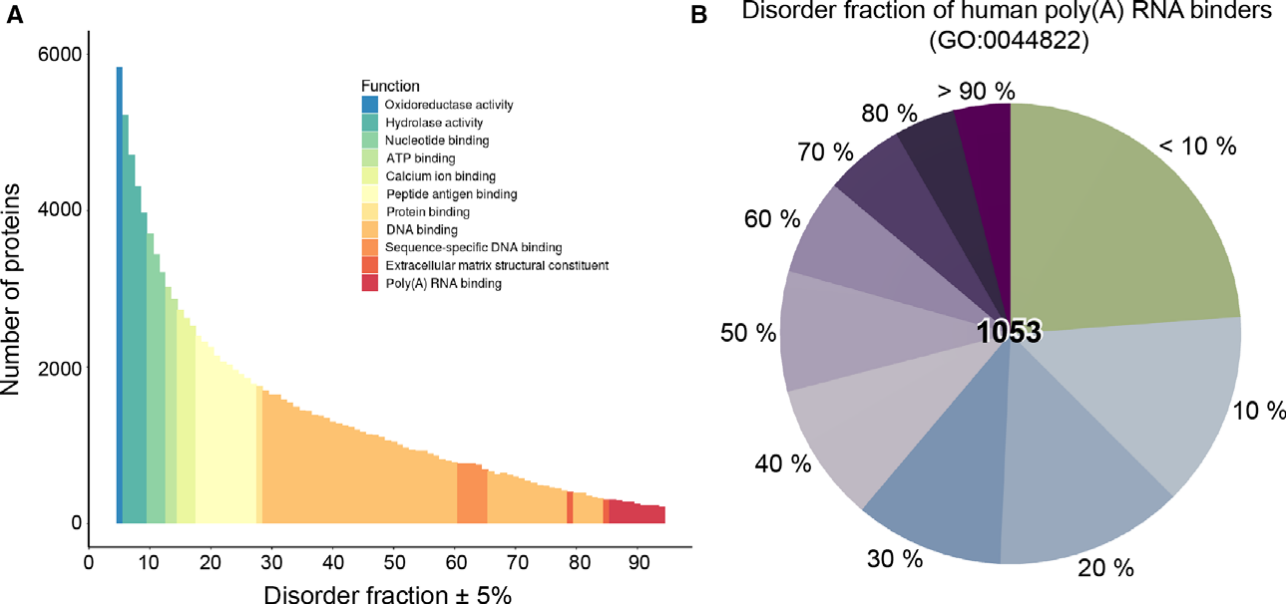
RNA binding and protein disorder. (A) Distribution of the degree of structural disorder among human proteins according to IUPRED 42, with the most enriched GO functional term for each bin indicated in color; (B) Structural disorder among poly(A) RNA‐binding proteins in human according to IUPRED 42. Figure 1A was reproduced with permission (Oxford University Press) from Ref. 13.

Our understanding of biology at the molecular level is transforming rapidly and central to this is a radical reappraisal of the importance and ubiquity of RNA–protein interactions 1, 2, 3, 4, 5, 6. From gene expression regulation to RNA processing and decay to protein localization, it is now clear that many cellular processes are unimaginable without direct, specific interactions between RNA and RNA‐binding proteins (RBPs) 1, 2, 3, 4, 5, 6, 7. A good example in this regard is the case of mRNAs and their interaction networks. Namely, recent proteome‐wide experiments utilizing UV‐crosslinking and mass spectrometry have led to hundreds of newly identified proteins, which interact directly with mRNAs, but do not contain any known RNA‐binding domains (RBDs) 2, 5, 6, 8, 9, 10, 11, 12. For example, 40% of the 570 identified yeast mRNA‐binding proteins lack well‐defined RBDs, are not associated with any presently known functions in RNA biology and even include different metabolic enzymes and transcription factors 10. Importantly, many such ‘enigmRBPs’ contain repetitive, low‐complexity sequence regions and are intrinsically unstructured i.e., disordered 2, 5, 6, 8, 9, 10, 11, 12.^1^ A systematic Gene Ontology (GO) analysis of the dependence between the calculated degree of structural disorder and functional enrichment clearly shows that ‘poly(A) RNA binding’ is the most enriched function among highly disordered proteins (>80%) in human (Fig. 1A) 13. In general, RNA/DNA binding functions feature strongly among the highly disordered proteins (Fig. 1A). The reverse is also true: ~30% of all mRNA‐binding RBPs in HeLa cells, for example, have one half or more of their residues in an unstructured state 5, 10 (Fig. 1B). Traditionally, structural disorder in proteins has not only been linked with transcription, chromatin modification, and signaling 14, 15 but is also key for the assembly of phase‐separated organelles such as P‐bodies and stress granules, major sites of RNA processing and storage 16, 17, 18, 19, 20. Although conserved flexibility and disorder are important for RNA–protein binding, the fundamental principles behind such interactions remain largely unexplored. As a consequence, the computational tools to predict binding between RNA and unstructured proteins are scarce 21. The present review focuses on the recent work on RNA–protein interactions in an unstructured context with a particular focus on how the specificity in such interactions may be determined.

## Cellular context of RNA interactions with unstructured RBPs

Unstructured RBPs are closely involved at all stages of the life cycle of different RNAs in the cell. In the case of mRNAs and long noncoding RNAs (lncRNAs), this includes transcription, processing by the splicesosome and the exon junction complex, nuclear export, translation at the ribosome, and decay (reviewed in 5 and 22). Importantly, a series of recent studies have reported instances of phase separation in the cytoplasm and nucleoplasm, a process similar to lipid domain (e.g., so‐called ‘rafts’) formation in the membrane, which in aqueous environment results in the formation of liquid droplets (reviewed in 20, 23, and 24). These droplets are typically rich in proteins and RNA and define nonmembrane‐bound cellular compartments such as nucleoli, P‐bodies, stress granules, and Cajal bodies. They are also known to be the sites of mRNA storage, processing, and decay. What is critical here is that it has been shown that both multivalency (i.e., existence of many low‐affinity binding sites) and the presence of low‐complexity, disordered regions in proteins may be required for the formation of such phase‐separated compartments 19, 23, 25, 26. Moreover, such compartments are known to preferentially attract single‐stranded nucleic acids and are stabilized in their presence 25, 27. For these reasons, it is likely that understanding of RNA interactions with disordered proteins and their contextual dependence may contribute directly to our understanding of the structure, assembly, and function of phase‐separated cellular compartments as well. Conversely, this also suggests that it is critical to study such interactions in the context of crowded, dehydrated, low‐dielectric environments similar to those present in P‐bodies or stress granules, where RNA molecules may also be in a single‐stranded, unstructured form.

## Lack of 3D organization motivates sequence‐based analysis

**Figure 2:**
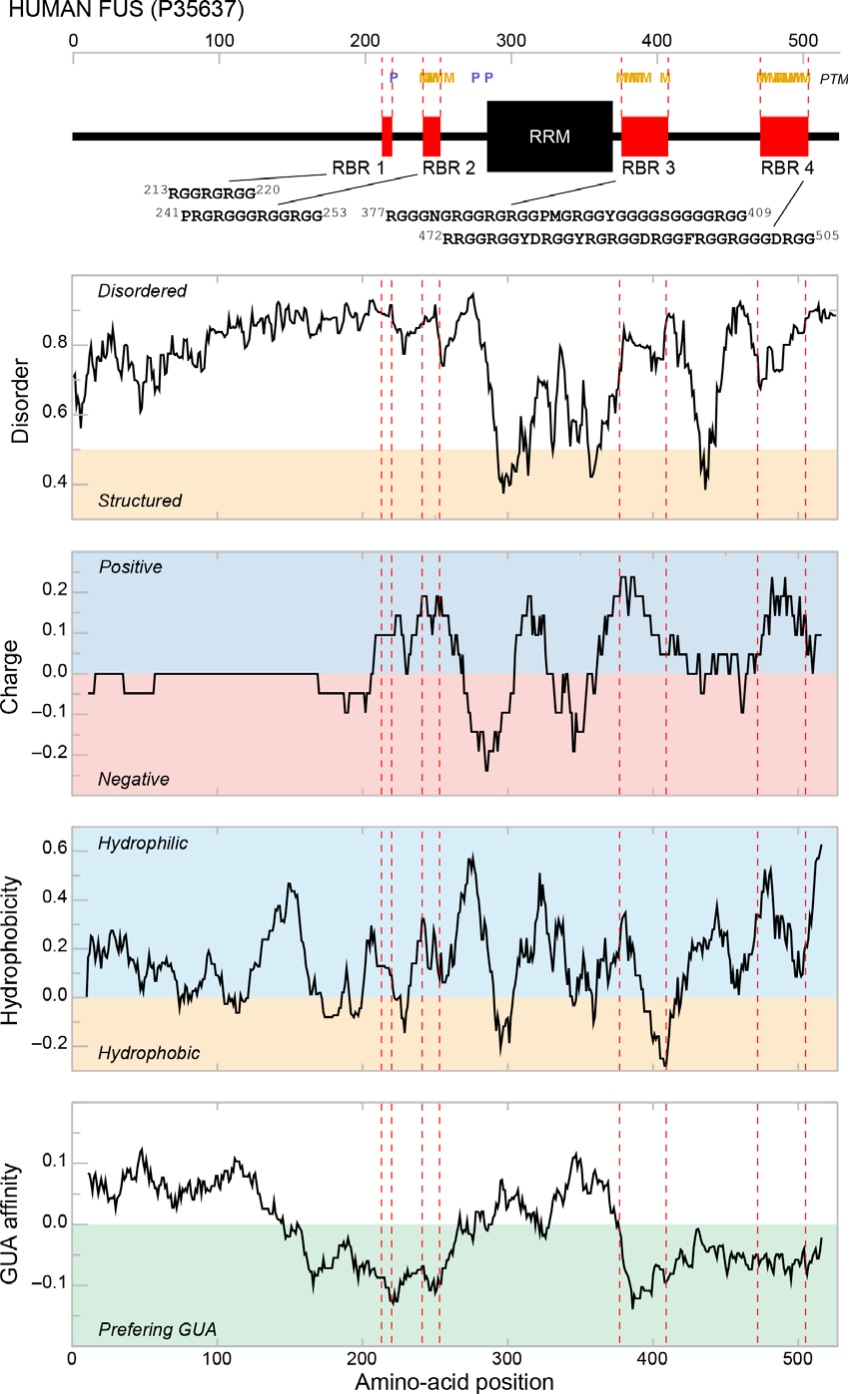
1D physicochemical profiles of FUS. Disorder, charge, hydrophobicity and GUA‐affinity profiles of human FUS in relation to its domain structure. The charge, hydrophobicity and GUA‐affinity profiles were determined by using a running‐average window of 21‐residues.

Due to diminished structural constraints, the properties of intrinsically unstructured/disordered proteins (IDPs) depend much more on their linear sequence features than in the case of folded proteins 14, 15, 28. For example, accurate prediction of protein disorder using sequence information is almost routine nowadays 28, 29. In general, the structural, dynamical, and functional characteristics of IDPs can in many cases be successfully related to the linear distribution of different physicochemical properties of individual residues and/or short fragments along their sequence, that is, 1D physicochemical profiles. As an illustration, we present in Fig. 2 several such 1D profiles obtained for Fused‐In‐Sarcoma (FUS) protein 26, 30, 31, 32 including its disorder probability, charge density, hydrophobicity, and GUA‐affinity profiles. FUS is an abundant nuclear protein involved in mRNA transcription, processing, and transport 30, 31, 33, 34, 35, 36, 37 and implicated in the pathophysiology of amyotrophic lateral sclerosis and frontotemporal lobar degeneration 37, 38. Importantly, FUS contains four highly disordered RNA‐binding regions (RBR) including RGG/RG domains 5, whose arginine residues are known to be the targets of arginine methyl transferases 39. This post‐translational modification has been shown to modulate the nuclear transport of FUS 40 as well as RNA‐binding activity of another RNA‐binding IDP FMRP 41.

A distribution of disorder probability along the FUS sequence, that is, its disorder profile as calculated in this case by IUPRED 42, gives one a possibility to identify unstructured regions within the protein and match them with the known RBRs. For instance, repetitive linear sequence elements required for RNA binding, like RS and RGG/RG domains, reside in highly unstructured regions and/or the flanking regions of structured RNA‐binding domains (e.g., RRM) as was also shown for a number of RNA‐binding IDPs (see Järvelin *et al*. 5 for detailed review of disorder organization in 46 different well‐characterized RNA‐binding IDPs). The FUS RRM in particular is surrounded by two RGG/RG domains (RBR 2 and 3, Fig. 2). Such an organization enables a synergy between disordered and structured RNA‐binding domains and can increase the RNA affinity of the RRM in question as compared to an isolated one. For example, it has been shown recently that in FUS the flanking RGG/RG domains actually enable the RNA‐binding activity of its RRM 32. While RRMs typically exhibit highly conserved sequence features, the flanking regions can also display sequence conservation and more importantly—disorder conservation 29. Using DisCons, a server for the analysis of disorder score conservation in multiple sequence alignment 43, Varadi and coworkers have shown that disorder conservation is especially pronounced for the residues at RNA‐binding interfaces.

A 1D distribution of charged residues along protein sequence gives one the possibility to identify prominent negatively or positively charged regions. Interestingly, the FUS RRM together with the flanking RBRs displays a prominent alternation in the net charge (Fig. 2). Many IDPs are polyampholytes and exhibit simultaneously positively and negatively charged regions, whereby their distribution within the sequence shapes protein conformational behavior 44 and can also modulate phosphorylation patterns within the disordered regions 45. The alternating sequence of net charge has also been shown to be an important determinant in phase‐separated droplet formation involving DDX4 RNA‐helicase 25. A distribution of hydrophobic residues along an IDP sequence is another important property which affects liquid phase separation, due largely to the contribution of aromatic residues to multivalent interactions with RNA and other proteins (e.g., π–π and cation–π), as shown for a number of RNA‐binding IDPs including FUS 19, 23, 25, 26, 46, 47, 48. Interestingly, while the FUS RRM is prominently hydrophobic, its RBRs display a different level of hydrophobicity depending on the neighboring sequence context (Fig. 2; the FUS hydrophobicity profile was determined by using a consensus hydrophobicity scale Factor I 49). This could contribute to a slightly different sequence specificity of FUS RBRs and the preference of its RRM to interact with the relatively more hydrophobic ADE‐rich sequences 32. Finally, in order to map directly the RNA‐binding specificity along the FUS sequence, we plot its 1D guanine (GUA) affinity profile as derived by using a knowledge‐based nucleobase/amino acid side‐chain affinity scale (see below) and the formalism described elsewhere 50. Here, one can see that the disordered RBR regions perfectly match the peaks of GUA‐affinity, which is in line with an experimental observation of the general preference of these regions for GUA‐rich sequences 5, 32 (N.B. following the standard thermodynamic convention, the low ΔG values correspond to high affinity and *vice versa*). This also agrees with the analysis of the distribution of GUA‐preferring amino acids in structured proteins, which shows that they are mostly enriched in unstructured loop regions 51.

## Sequence‐based prediction of RNA–protein interactions

Computational prediction of RNA–protein interactions in an unstructured context is still relatively underdeveloped due in part to our lack of understanding of the basic physicochemical principles at play and a general lack of high‐resolution information when it comes to unstructured partners. This situation, however, will likely change in the near future and the lessons learned and the methods developed when it comes to RNA–protein interactions in general will likely be important in the case of structural disorder as well. Most modern methods for predicting RNA–protein binding from sequence information, physicochemical properties of individual RNA and protein building blocks, or global RNA–protein characteristics are based on machine learning strategies. In particular, predictive models are trained on the known interactors by using features such as composition, hydrophobicity, and evolutionary information 52, 53, 54, 55. An early such approach, developed by Pancaldi and Bahler, is based on support vector machine (SVM) and random forest (RF) formalisms and uses the known RNA and protein features as predictors, including *inter alia* GO terms, protein localization, and chromosome position information 56. A comparable approach developed by Dobbs and coworkers, also using SVM and RF approaches, showed that using just sequence information as descriptors may result in a significantly better prediction 57. Chen and coworkers employed an alternative approach and used an extended naïve Bayes approach on sequence composition data 58. In contrast, Tartaglia and coworkers put a major focus on the physicochemical properties and trained their catRAPID model on features such as secondary structure propensity, hydrogen bonding, and van der Waals interactions 59, 60. Neural networks have also been applied to identifying RNA‐protein interactions, following their overwhelming success in areas such as image or speech recognition and natural language processing. DeepBind, for example, uses a neural network trained on several experimental datasets including RNAcompete, ChIP‐seq, and HT‐SELEX experiments and performs well over a wide range of metrics 61. Qu and coworkers have shown the benefits of a deeper network in the rather similar case of DNA binding: for an extensive realistic dataset, an accuracy of 94.2% could be achieved 62. RCK, on the other hand, uses k‐mers to evaluate the binding propensity and slightly outperforms DeepBind, at least on the RNAcompete dataset 63, 64. Finally, RNAcontext, although somewhat outdated, still performs well in comparison to RCK and DeepBind, sometimes even outperforming the two. In this approach, both RNA sequence and secondary structure information are used to accurately predict binding to RBPs 65.

Although they tackle an important and difficult problem, the above methods still frequently suffer from a limited accuracy, a lack of general applicability, and the fact that only a few of them 21, 50, 53, 59 are fundamentally steeped in basic physicochemical principles. The latter criticism is probably the most important downside of the machine learning approaches: they frequently do not allow for a deeper insight into the physicochemical underpinnings of RNA–protein interactions. Another limitation concerns the availability of experimental data to train these models on. For instance, the only existing method for predicting RNA binding to IDPs, DisoRDPbind 21, was trained on a set of only 14 annotated RNA‐binding proteins. Here, we would like to argue that it may in many cases be more advantageous to approach this problem purely from a physicochemical perspective and exploit the intrinsic affinities between nucleobases and amino acids as a foundation for predicting the interaction between longer biopolymers. This approach seems promising especially in the context of single‐stranded RNA interacting with unstructured proteins, given that in those cases the dependence on the properties of basic building blocks is likely most pronounced.

## Nucleobase/amino acid affinities as a basis for understanding RNA–IDP interactions

The recent findings about the mode of interaction between RNA and IDPs highlight the relevance of predicting and understanding such interactions from the perspective of first principles. For instance, it is known that short linear motifs with <10 residues are one of the primary ways of how IDPs interact with partners 22, 28. Moreover, even for IDPs that fold upon binding, the participating regions comprise mostly local, 10–70 residue segments 22, 28. On the other hand, RNA‐binding sites for proteins also typically include just a few nucleotides organized in single‐stranded, linear stretches 63, 66. For example, RNAcompete studies have shown that most RBPs bind single‐stranded RNAs with <10 nucleotides, and none absolutely requires a defined RNA secondary structure 63. Hence, one may expect that the principles of RNA interactions with IDPs or the unfolded states of otherwise folded proteins can be deduced by examining structural and thermodynamic aspects of interactions between individual nucleobases and amino acids. With diminished structural constraints, the behavior of long polymers can more easily be related to their constituent building blocks, a possibility that has remained unexplored until recently.

**Figure 3:**
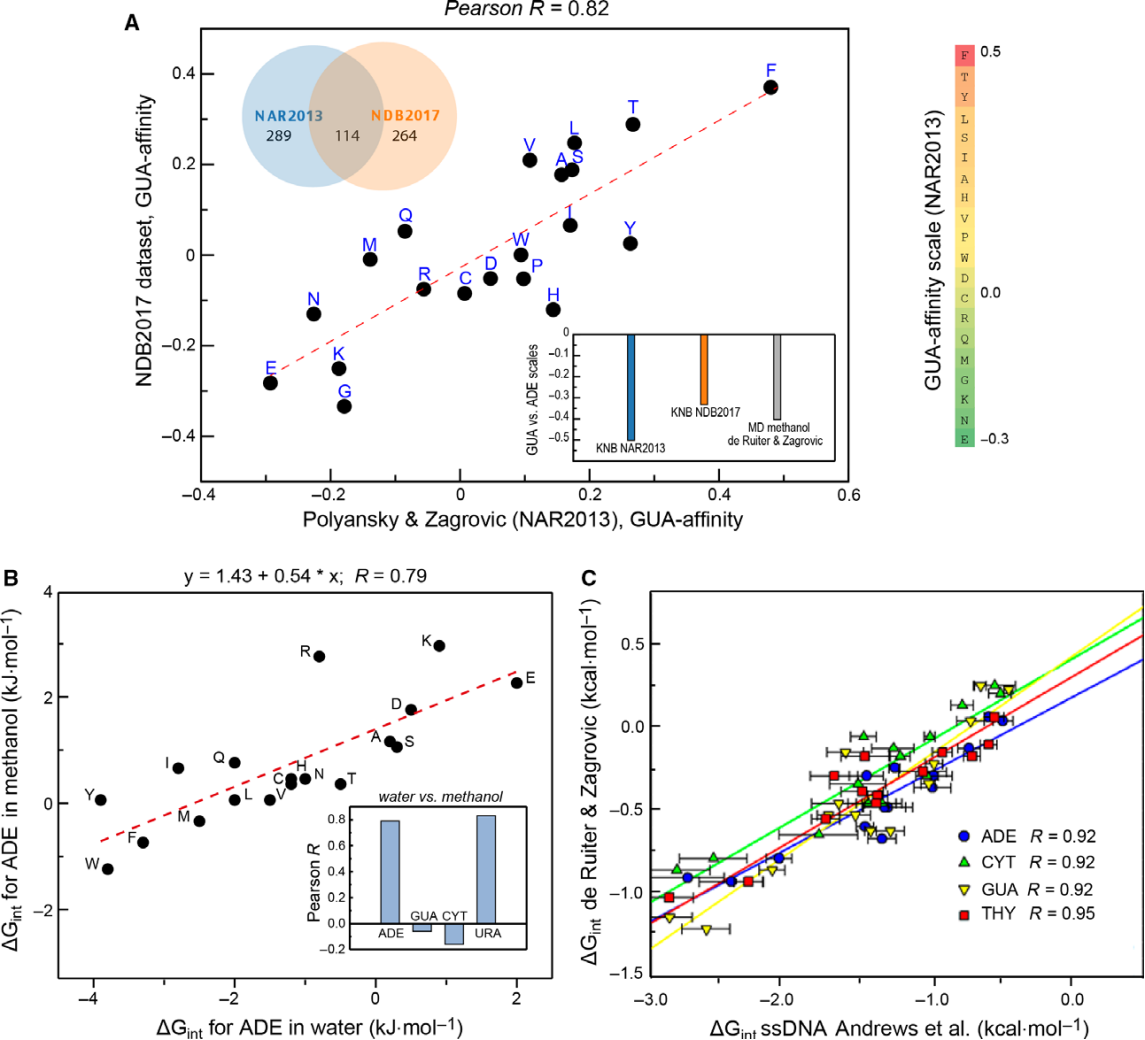
Robustness of affinities between nucleobases and amino acid side chains. (A) A close correlation between the knowledge‐based (KNB) scales of GUA/side‐chain affinity derived from two largely independent sets of structures of RNA–protein complexes (NAR2013 50 and NDB2017). The NDB2017 set was generated using a representative dataset from the Nucleic Acid Database (http://ndbserver.rutgers.edu) 110 with the resolution cutoff of 2.5 A using the identical method as described by Polyansky *et al*. 50. The overlap between the two sets is given by the Venn diagram. Inset: anticorrelation between the GUA and ADE side‐chain affinity scales is observed for two sets of knowledge‐based scales (NAR2013 50 and NDB2017) and the MD‐based scales of nucleobase–amino acid affinity in methanol 81. The bars indicate the Pearson R coefficients between the GUA and ADE scales. (B) ADE and URA amino acid‐binding free energy scales are up to a constant largely insensitive to local dielectric constant, while those for GUA and CYT strongly depend on it (Pearson Rs given in the inset) 81. (C) Andrews *et al*. 80 nucleobase–amino acid‐binding ΔG scales (*x*‐axis) correlate closely with de Ruiter *et al*. scales 81 (*y*‐axis) Figure 3C was reproduced with permission (American Chemical Society) from Ref. 80.

Significant progress in understanding nucleobase–amino acid interactions has over the years been made using computational approaches 50, 67, 68, 69, 70, 71, 72, 73, 74, 75, 76, 77, 78, 79, 80. For example, analysis of 3D structures of RNA– or DNA–protein complexes has yielded the relative binding preferences of nucleobases and amino acids together with a geometric and energetic characterization of their interactions 50, 67, 68, 72, 75, 76, 77, 78, 79. Despite a limited amount of statistics that could be extracted from the analysis of 3D complexes 50, the obtained amino acid preferences for GUA and adenine (ADE) are significantly robust and reproducible when it comes to the scale values and a moderate anticorrelation between the GUA and ADE scales as shown for different sets of RNA–protein complexes (Fig. 3A). Also, *ab initio* methods have been used to study the quantum‐mechanical aspects of such binding, including hydrogen bonding 71, π–π 73, 74 and cation–π interactions 70. Finally, the binding free energy maps for several amino acids and DNA base pairs have also been reported 69. While these early studies have typically either focused on a few bases and amino acids only or have simply been insufficiently quantitative, recently there appeared several computational studies with a more comprehensive outlook. For example, our recent analysis of the absolute binding free energies between all standard RNA–DNA nucleobases and amino acid side‐chain analogs in different solvents 81, 82 and their dependence on the local dielectric properties is, to the best of our knowledge, the first example where this key property has been determined within a single, self‐consistent framework. Interestingly, the dielectric properties of the environment tune the affinity of amino acid side chains for GUA and cytosine (CYT), while having little or no effect on ADE and uracil (URA) scales, as shown by using umbrella‐sampling simulations of individual nucleobases and amino acid side‐chain analogs in water and methanol (Fig. 3B) 81, 82. These results are especially important when it comes to understanding the basic principles of RNA–IDP interactions in the context of liquid RNA–protein granules characterized by a reduced dielectric constant as compared to bulk water. Importantly, the obtained affinities in water were found to be in an excellent agreement with the results of a subsequent independent analysis by Elcock and coworkers who have performed explicit‐solvent MD simulations of long single‐ and double‐stranded DNA molecules in heterogeneous aqueous mixtures of amino acids using a different MD force field 80 (Fig. 3C). The latter analysis also provided a comprehensive dissection of the salt dependence of nucleobase–amino acid interactions and the contribution of DNA sugar and phosphate groups to binding. Moreover, the nucleobase–amino acid affinity scales were also derived based on a simulated partitioning of amino acids between nucleobase‐rich phases and water 83, 84. Finally, Vondrasek and coworkers have used the known PDB structures of DNA–protein complexes together with molecular mechanics and DFT‐D *ab initio* calculations to estimate the binding preferences between all 20 natural amino acids and the four DNA bases 79.

When it comes to experimental determination of nucleobase–amino acid‐binding preferences, only limited progress has been made over the years. Akinrimisi *et al*. 85 and Thomas *et al*. 86 have studied spectroscopically the solubility in water of several amino acids in the presence of either purines or different nucleosides, respectively. In the same way, Thomas *et al*. 86 have determined binding constants for several nucleoside and amino acid pairs. On the other hand, Woese *et al*. have evaluated chromatographically the interaction propensities of amino acids and different pyridine derivatives in water 87, 88. Finally, several groups have studied interactions between different nucleotides and polyamino acids, focusing typically on polylysine or polyarginine peptides 89, 90, 91. The scarcity of the experimental studies in this context can in part be attributed to the low solubility of bases and some side chains as well as the weak interaction strengths involved. For example, our recent work suggests that only a handful of nucleobase side‐chain affinities exceed 1 k_B_T 81, 82. As discussed below, however, we see a systematic experimental determination of such affinities as one of the major open challenges for the future.

## Nucleobase–amino acid affinities reflect the genetic code organization

As discussed above, RNA–protein interactions in an unstructured context are a ubiquitous feature of modern biological systems. However, they also provide an important perspective for understanding the establishment of the RNA–protein relationship in primordial systems in which structural disorder is likely to have been even more pervasive 92. This, in particular, concerns the central aspect of the whole RNA–protein relationship: the process of translation and the genetic code 93, 94. Namely, with minor variations, the genetic code is universally conserved and it, without exaggeration, represents the very point where biological phenotype and genotype meet. However, despite 50 years of effort, the nature of the driving forces behind its establishment has remained largely unknown. Over the years, multiple theories have been proposed in this regard with varying levels of evidence 94, 95, 96, 97. Of relevance here, the ‘stereochemical hypothesis’ suggests that the key feature of ancient translation was a direct interaction between codons and amino acids they code for 87, 88, 94, 98. Although specific binding of isolated codons and their cognate amino acids has never been observed, analysis of amino acid‐binding RNA aptamers 98 and RNA–protein interactions in the ribosome 99 has revealed that not only some codons but also anticodons, preferentially colocalize with their cognate amino acids. Importantly, early support for the hypothesis came from Woese and coworkers who analyzed the interaction preferences between amino acids and pyrimidine mimetics pyridines 87, 88, 94, 100 (see also above). They showed that amino acids with a similar propensity to interact with pyridines also have similar codons.

**Figure 4:**
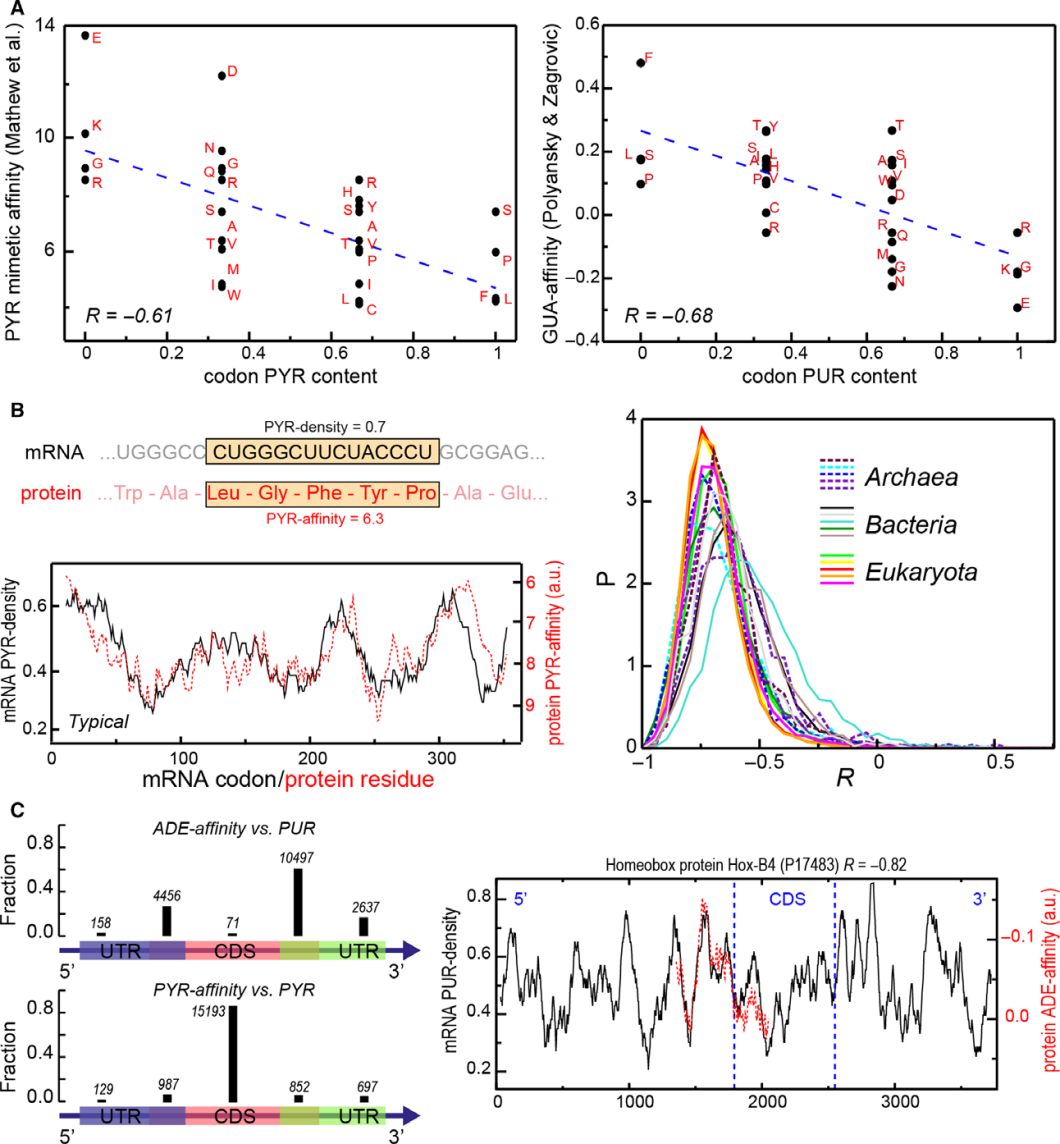
The complementarity hypothesis. (A) Codon PYR content correlates with the cognate amino acid affinity for PYR mimetics 100, while codon PUR content correlates with the cognate amino acid affinity for GUA 50. (B) Right: profile calculation method together with a typical pair of mRNA PYR density and protein PYR‐mimetic affinity profiles in human; left: Pearson Rs for mRNA PYR density/protein PYR‐mimetic affinity profiles in 15 species. (C) Left: Location of top matches for human mRNAs and cognate proteins, including UTRs and transition regions (violet/olive) for mRNA PUR density/protein ADE affinity, and mRNA PYR density/protein PYR affinity cases. Numbers of top matches are given above bars. Right: An example of a top match between mRNA PUR density and cognate protein ADE affinity. Figures 4A and 4B were reproduced with permission (Oxford University Press) from Ref. 102.

A common feature of most studies of the code's origin has been their focus on individual codons and amino acids only 93, 94, 101 with little attention paid to the properties of longer biopolymers. However, any biases present at the level of individual groups may get cooperatively amplified in such cases, facilitating their detection. Moreover, if the stereochemical hypothesis is indeed true, then the genetic code could also be seen as a key for understanding RNA–protein interactions in general. Following this paradigm, we have recently explored the link between the physicochemical properties of mRNAs and the proteins they encode 102, 103 and have made a surprising discovery. First, using both experimental and computational nucleobase–amino acid affinity scales, we could show that the nucleobase content of a given codon is directly related to the affinities of its cognate amino acids for the respective nucleobases (Fig. 4A). For example, the codon PYR content correlates with the PYR‐mimetic affinity of the cognate amino acids with a Pearson R of −0.61, while the codon PUR content correlates with the knowledge‐based GUA‐affinities of the cognate amino acids with a Pearson R of −0.68 (Fig. 4A). What is more, this relationship was even further magnified in the context of complete biopolymers. A pyrimidine (PYR) density profile of an mRNA coding sequence can be obtained as a running average of the PYR content of its codons (Fig. 4B). Similarly, a PYR‐affinity profile of the cognate protein can be estimated by weighting its sequence by the amino acid propensities to interact with PYR mimetics as captured in Woese's experiments or subsequent simulations 88, 100. The remarkable finding is that for most mRNA–protein pairs the two profiles match closely when aligned 102, 103. For example, the median Pearson correlation coefficient R between the two profiles over all human pairs is *R* = −0.74 (note that all affinity scales are defined such that negative Rs mean matching 102, 103).

This is unexpected and potentially far‐reaching: although mRNAs and their cognate proteins are completely different biopolymers, they exhibit a strong, quantitative complementarity in that the density of a particular type of groups in one of them can be accurately predicted from the affinity profile for similar groups in the other one. Importantly, we could show that this finding is statistically extremely robust and holds equally well for organisms from all three domains of life (Fig. 4B) 102, 103. Moreover, we have confirmed these findings for biologically relevant PYRs as well as extended them to purines (PURs) by using knowledge‐based nucleobase–amino acid affinities derived from structures of RNA–protein complexes 50, 103. For example, mRNA PUR density profiles quantitatively match GUA‐affinity profiles of cognate proteins, with a median of *R* = −0.80 in human 50, 103. Notably, protein ADE affinity profiles exhibit a reverse property in that they match the PYR density of cognate mRNAs. We have shown that this stems from biosynthetically more complex amino acids that are thought to have entered biology later 50, 103. Finally, we have fully corroborated the above findings by using affinities derived by orthogonal approaches including umbrella‐sampling MD simulations 81, 82 or modeling of partitioning experiments 83, 84.

**Figure 5:**
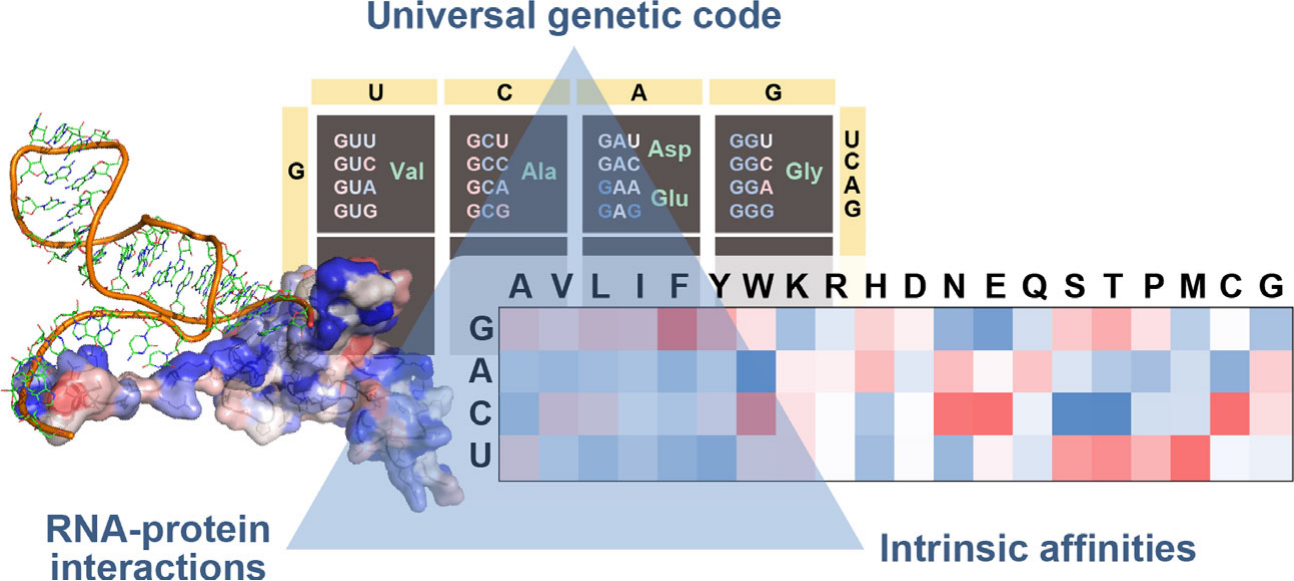
The universal genetic code as the Rosetta stone for understanding RNA–protein interactions.

The above results provide support for the stereochemical hypothesis of the origin of the genetic code, but they emphasize the importance of an extended, polymeric context in which the relatively weak affinities of individual building blocks can be amplified. Moreover, these findings support a novel hypothesis that in the unstructured state, mRNAs and the proteins they encode may be complementary to each other and bind in a coaligned manner, whereby the complementarity level is negatively regulated by mRNA ADE content [50, 51, 81, 82, 83, 84, 103, 104, 105]. Since compositional matching is seen for primary sequence profiles, we expect that the strongest interactions will occur if the partners are unstructured, yielding dynamic, multivalent, liquid‐like complexes: in addition to IDPs, the hypothesis applies equally well to the unfolded states of otherwise folded proteins 51. Importantly, the coarse‐grained nature of the complementarity hypothesis allows one to extend it to interactions between unstructured proteins and nucleic acids other than their cognate mRNA coding regions 102, 103. The key point is that a physicochemical view of biomolecular sequences provides a measure of both binding propensity in an unstructured context and evolutionary relatedness for different RNA and protein molecules. In this sense, the above findings could be interpreted as general rules for understanding RNA–protein interactions: amino acids whose codons are enriched in PYR also display proteome‐wide tendencies to be specific for PYR or ADE, while polar amino acids, encoded by PUR, predominantly interact with GUA (Fig. 4A). Moreover, these rules could be applied in noncoding situations as well. This can be illustrated in the case of noncoding 5′ and 3′ untranslated regions (UTRs) of mRNAs. For example, for hundreds of human mRNA sequences, the top match between the mRNA nucleobase density profiles and their cognate proteins’ nucleobase‐affinity profiles is observed in the UTRs or in the transitional regions including a UTR and a part of the coding sequence (Fig. 4C). In fact, for mRNA ADE density profiles, the majority of mRNAs and their cognate proteins fall into these categories. This is well illustrated in the case of HoxB4 mRNA and its cognate protein, where the top matching region includes a significant part of the 5′ UTR (Fig. 4C). As a whole, the above findings suggest that the structure of the universal genetic code reflects, in part, the binding specificity between nucleobases and amino acids and, conversely, support an exciting possibility that the universal genetic code may be seen as a key for understanding RNA–protein interactions in the unstructured context and beyond (Fig. 5).

## Significance and outlook

An improvement in our understanding of the RNA–protein interactions in the unstructured context, including the ability to predict binding sites and relative affinities, would represent a major step forward from both fundamental and practical perspectives. Currently, most of our knowledge of RNA–protein interactions concerns structured RNA‐binding motifs and, consequently, computational methods for predicting RNA‐protein interactions in a physicochemically and structurally realistic manner are necessarily limited and biased by such knowledge [21, 53, 54, 55, 59, 106]. Similarly problematic, the sequence‐based computational approaches for predicting RNA–protein interactions typically involve machine learning strategies and only indirectly rely on or help further understand the microscopic physicochemical principles behind such interactions 53, 54, 55. While experimental approaches toward enumerating and characterizing RNA interactions with IDPs are making significant advances [2, 5, 8, 9, 10, 29, 63], they are limited by the fact that the microscopic, high‐resolution features of such complexes are largely beyond the reach of modern structural biology methods. It is, therefore, imperative to explore the existence of general, novel physicochemical principles behind such interactions. Our ‘complementarity hypothesis’ may provide one such principle. In general, physicochemical complementarity is one of the most powerful paradigms used to explain biological function at the molecular level. Complementarity between DNA strands is the key element behind gene duplication, antibody–antigen complementarity guides immune response, while enzymes cannot be understood without the complementarity between active sites and reaction transition states. Therefore, it is potentially extremely far‐reaching that RNA and protein sequences, including both cognate mRNA–protein pairs, as shown in our recent work 50, 51, 81, 83, 84, 103, 104, 105 and those not connected by coding, as illustrated above, would exhibit such a robust compositional complementarity. This finding simply demands detailed exploration and full explanation and we see it as a major challenge for the future. Having said this, the range of validity and general applicability of the ‘complementarity hypothesis’ remain unclear: for example, the fact that the contour length of a typical mRNA coding region is approximately five times longer than the contour length of its cognate protein suggests that any physical realization of putative complementary binding must negotiate a significant spatial challenge. However, the hypothesis provides a well‐defined, testable framework for relating the fundamental physicochemical properties of nucleobases and amino acids with the interactions of complete RNAs and proteins in an unstructured context. Moreover, the hypothesis promotes a way of looking at RNA–protein interactions that could be of practical importance regardless of whether the hypothesis itself turns out to be true or not. In particular, viewing RNA and protein sequences as physicochemical entities, with specific properties and local interaction propensities, presents a powerful paradigm for linking the speed and power of standard bioinformatic techniques with the atomistically realistic rigor. The consequences of this line of research potentially concern all canonical areas of bioinformatics and computational biology ranging from sequence comparison to multiple sequence alignment to building of phylogenetic trees.

There are a number of important open challenges regarding the RNA–protein interactions in an unstructured context. Here, we outline what we believe are the three most relevant and fundamental such challenges that have thus far, somewhat surprisingly, escaped wider attention. We firmly believe that these issues hold a key to a deeper understanding of RNA–protein interactions in an unstructured context, but could also prove to be essential on a much wider scale.

### CHALLENGE 1: experimental determination of nucleotide–amino acid affinities

Nucleotides and amino acids are arguably the most fundamental building blocks in all living matter. Moreover, the specificity in interactions between nucleic acids and proteins is directly shaped by the specificity in interactions between these individual building blocks. Considering this, it is remarkable that the pioneering, yet incomplete experimental studies aimed at determining the affinities between individual nucleotides and amino acids, that is, their fragments 85, 86, 87, 88, 89, 90, 91, have not been extended since the 1960s and 1970s when they were first performed. There currently exists no self‐consistent, experimentally determined table of absolute or, for that matter, relative binding ΔGs between the 5 standard RNA–DNA nucleotides (i.e., nucleosides/nucleobases) and the 20 standard amino acids (i.e., amino acid side chains). As discussed above, there is a growing body of computational/theoretical work in this regard 50, 77, 78, 79, 80, but the experimental contributions remain surprisingly incomplete. A part of the reason for this are the low solubilities of some of the groups and/or the low affinities involved, but we are firmly convinced that these difficulties can be successfully addressed with the aid of clever experimental strategies using, for example, affinity chromatography, NMR, microscale thermophoresis, or high‐precision ITC. A natural extension of such studies would be a careful dissection of the binding contributions of different fragments of individual nucleotides and amino acids, such as the sugar or the phosphate groups, as well as an analysis of the impact of post‐transcriptional nucleotide modifications and post‐translational amino acids modifications on the individual binding affinities. For example, by using a purely computational analysis, we have recently shown that deamination of adenine, one of the most biologically important post‐transcriptional nucleobase modifications, changes its interaction pattern with amino acids to that of guanine 107. It would be critical that such and similar studies be extended from an experimental side as well. One criticism may be that nucleotide–amino acid interactions will be severely context dependent and susceptible to local environmental influences (pH, ionic strength, dielectric constant, temperature etc.) and, therefore, too difficult to accurately pin down. While we share this apprehension, we are convinced that a systematic analysis of such influences is in order. Moreover, our computational results strongly suggest that certain robust patterns of behavior remain even under changing environmental conditions 81, 82. Such robustness, after all, must be there simply for the biological systems to be able to function in a stable way.

### CHALLENGE 2: analysis of sequence specificity in the formation of phase‐separated granules

Phase‐separated granules represent arguably the best, biologically relevant system for studying the interactions between RNA and proteins in an unstructured context 16, 17, 18, 19, 20. On the one hand, single‐stranded RNA and disordered proteins are ubiquitous constituents of such granules. On the other hand, the weak, multivalent, dynamic complexes formed by disordered RNA and proteins naturally lead to liquid–liquid phase separation that can also be well studied *in vitro*. In other words, phase separation and RNA–protein interactions in an unstructured context go hand in hand and should therefore be studied from a joint perspective: understanding of RNA–protein interactions in the unstructured state will provide critical information for the understanding of granule formation and *vice versa*. Importantly, a growing body of work has shown that the presence of RNA, and in particular single‐stranded RNA, markedly reduces the critical concentration required for the formation of phase‐separated protein granules 20, 25, 27. However, a major open question concerns the sequence specificity behind such effects. In most cases, the authors have used random RNA sequences and/or select homooligonucleotides, but there have been no fully systematic attempts at understanding how different RNA, that is, protein sequences influence each other in this regard. We predict that the impact of different RNAs on the formation of protein granules will follow the rules given by the universal genetic code 50, 102, 103. For example, we predict that GUA‐rich sequences will have a stronger effect on the phase separation of protein sequences containing mostly polar residues, while ADE/PYR‐rich sequences will have a stronger impact on the more hydrophobic protein sequences. In the context of the complementarity hypothesis discussed above, it would also be particularly interesting to systematically study the impact of cognate mRNA–protein pairs when it comes to granule formation. These studies should go hand in hand with the determination of the individual binding affinities between nucleotides and amino acids and their environmental dependence as outlined in the first challenge above. For example, phase‐separated granules are known to be partially dehydrated, low‐dielectric environments in which the relative nucleotide–amino acid affinities may follow different rules than in the more aqueous environments. Our umbrella‐sampling calculations have shown that in methanol, a solvent with a significantly lower dielectric constant as compared to water, the affinity of GUA for the negatively charged Asp and Glu side chains increases multiple‐fold as compared to that in water 81, 82. Such and similar analyses will be necessary for the full understanding of microscopic driving forces behind the formation of phase‐separated granules. Conversely, phase‐separated granules will provide the proper biological context for studying the intricacies of the binding preferences between individual nucleotides and amino acids and RNA–IDP interactions in general.

### CHALLENGE 3: development of computational tools for the prediction/analysis of RNA–protein binding in an unstructured context

The third open challenge concerns the development of computational frameworks for predicting the sites and the strength of interaction between RNA and unstructured protein regions that would be based on fundamental physicochemical principles. While the top‐down machine learning‐based approaches definitely have significant merit, the more physicochemically motivated, bottom‐up strategies could provide a deeper mechanistic insight and have a greater predictive power. When it comes to the interaction between single‐stranded RNAs and largely unstructured proteins, successful strategies could be based on the knowledge of the intrinsic interaction affinities between the individual building blocks of the two polymers, as discussed above. Presently, we do not have a clear prescription for how this could be implemented practically, but are motivated by a simple analogy. Namely, hybridization of two strands of DNA or folding of an RNA molecule can be well predicted from a simple thermodynamic quantification of Chargaff pairing rules and local stacking propensities 108, 109. Local affinities of nucleotides for each other, together with some understanding of the effect of local neighboring sequences and structures are often sufficient to predict, for example, the melting temperatures of duplexes or the folds of individual RNA molecules 108, 109. The point here is that the global structural and thermodynamic behavior of large nucleic acid molecules can be related to their linear sequence features and local interaction preferences. It is our hope that the rich world of RNA–protein interactions in an unstructured context could also in part be understood in such simple terms, which in turn would open up a myriad of different fundamental and applied possibilities. We are particularly excited by the possibility that the rules behind such interactions may actually be embedded in an ancient, already familiar codebook: the universal genetic code.

## Note

1 Throughout this text, we use the terms “unstructured” and “disordered” interchangeably when referring to a lack of well‐defined, persistent secondary or tertiary structure in macromolecules.
